# Belastungserleben pflegender Angehöriger während der Coronapandemie

**DOI:** 10.1007/s00391-022-02026-6

**Published:** 2022-02-15

**Authors:** Christina Theurer, Doreen Rother, Klaus Pfeiffer, Gabriele Wilz

**Affiliations:** 1 Institut der Psychologie, Abteilung Klinisch-Psychologische Intervention, Friedrich-Schiller-Universität Jena, Semmelweißstr. 12, 07743 Jena, Deutschland; 2 Robert-Bosch-Krankenhaus, Stuttgart, Deutschland

**Keywords:** Informelle Pflege, Pflegebelastung, Psychosoziale Belastung, SARS-CoV‑2, COVID-19, Informal care, Caregiver burden, Psychosocial burden, SARS-CoV‑2, COVID-19

## Abstract

**Hintergrund:**

Bereits vor der Coronapandemie waren pflegende Angehörige mit einer Vielzahl an Herausforderungen und Belastungen konfrontiert. Erste Onlineerhebungen zeigten eine Zunahme der Pflegebelastung während der Pandemie. Ergänzend hierzu wurde eine Erhebung zu Auswirkungen der Pandemie auf bereits belastete pflegende Angehörige in Deutschland durchgeführt.

**Methode:**

Im Rahmen einer Evaluationsstudie zu einem gestuften Beratungsansatz für hochbelastete pflegende Angehörige wurden von 165 Pflegenden quantitative und qualitative Daten zum Belastungserleben, zu Ängsten und Wünschen in der Coronapandemie bezüglich der Pflege erhoben. Die Auswertungen erfolgten deskriptiv und mittels qualitativer Inhaltsanalyse.

**Ergebnisse:**

Von den Angehörigen gaben 26 % starke Ängste an, sich mit SARS-CoV‑2 zu infizieren, 50 % befürchteten, dass die gepflegte Person erkranken könnte. Die Hälfte berichtete deutliche Auswirkungen auf den Pflegealltag (47 %) und nahm eine deutliche Erhöhung der Pflegbelastung (51 %) wahr. Als häufigste negative Auswirkungen auf den Pflegealltag wurden der Ausfall von Pflegedienstleistungen und ein Mangel an Zeit für sich selbst genannt. Dementsprechend wurde am häufigsten der Wunsch nach Unterstützung geäußert. Der Pflegegrad, das Alter der Pflegenden und die vorherige Nutzung des Pflegedienstes erwiesen sich als Prädiktoren für das durch die Pflegenden eingeschätzte Belastungserleben der Erkrankten.

**Diskussion:**

Die Ergebnisse verdeutlichen die negativen Auswirkungen der Coronapandemie auf die Belastung pflegender Angehöriger eindrücklich. Zur Bewältigung der komplexen Zusatzbelastungen benötigen Angehörige Angebote, die zu deren Teilhabechancen sowie zur Verbesserung der Versorgung und sozialen Teilhabe der Pflegebedürftigen während der Pandemie beitragen.

## Abstract (en)

**Background:**

Family caregivers are faced with a variety of challenges and burdens. Recent online studies showed that these burdens have increased even more during the corona pandemic. Here we conducted a low-threshold written survey to assess psychological distress in highly burdened family caregivers during the corona pandemic in Germany.

**Method:**

Both quantitative and qualitative analyses were conducted within the context of a tiered counselling intervention study for highly burdened family caregivers (*N* = 165) to assess their experience of burden, anxieties, worries and desires regarding care.

**Results:**

A quarter of all caregivers (26%) reported high fear of becoming infected with SARS-CoV‑2 and 50% feared their care recipient could get infected. Almost half of all caregivers reported (47%) that corona had a high impact on the care situation and increased the burden of caregiving (51%). A loss of professional support and less time for themselves were reported most often. The desire for additional support was frequently expressed. The level of care, the age of the caregiver and the previous use of care services were found to be predictors of the psychological distress experienced by care recipients.

**Conclusion:**

The results illustrate an increased burden related to caregiving during the corona pandemic. In order to cope with this complex additional burden, there is an even higher need for support to improve care and social participation for both care recipients and caregivers during the pandemic.

## Hintergrund

Aktuell werden von den 4,1 Mio. pflegebedürftigen Menschen in Deutschland 3,3 Mio. (80 %) überwiegend von ihren Angehörigen zu Hause versorgt [18].

Auch ohne die pandemiebedingten Einschränkungen sind pflegende Angehörige mit vielen Herausforderungen konfrontiert, die häufig mit körperlichen und psychischen Beeinträchtigungen einhergehen [15, 17, 19]. Erste Onlinebefragungen belegen eine Zunahme der Belastungen pflegender Angehöriger in Deutschland im Zuge der Coronapandemie [8, 16]. Eine Verschlechterung des eigenen Gesundheitszustandes wurde in einer Onlinebefragung von der Hälfte der pflegenden Angehörigen angegeben [16]. Die in dieser Untersuchung befragten Angehörigen (< 67 Jahre) pflegten Personen aller Altersgruppen (≥ 60 Jahre: 45 %). Für ältere Pflegende kommt erschwerend hinzu, dass sie neben den Gepflegten häufig selbst ein erhöhtes Risiko (z. B. aufgrund eigener Grunderkrankungen, Immunseneszenz) für schwere und tödliche COVID-19-Verläufe haben [14, 21].

Zur Entlastung pflegender Angehöriger in der Coronapandemie wurden durch das Bundeskabinett Akuthilfen beschlossen. Hierzu gehören erweiterte Leistungen bei Pflegezeit und kurzzeitiger Arbeitsverhinderung sowie flexiblere Gestaltungsmöglichkeiten bei der Pflege- und Familienpflegezeit [5]. Für ein besseres Verständnis, welche Entlastungen pflegende Angehörige darüber hinaus benötigen, sind differenzierte Untersuchungen der Veränderungen ihrer Pflegesituationen und ihres Belastungserlebens notwendig.

Vor diesem Hintergrund wurden Teilnehmende einer Interventionsstudie für hochbelastete pflegende Angehörige im Rahmen einer Zusatzbefragung zu Auswirkungen der Coronapandemie auf ihre Pflege- und Lebenssituation befragt. Die von ihnen gepflegten Personen waren mindestens 60 Jahre alt. Ergänzend zu den bisherigen Onlinebefragungen sollten mittels einer Offlineerhebung auch pflegende Angehörige mit geringem bzw. ohne Internetzugang oder -nutzung erreicht werden. Zudem sollten potenzielle Prädiktoren des Belastungserlebens der pflegenden Angehörigen und der Pflegebedürftigen untersucht werden.

## Material und Methoden

### Stichprobe

Die vorliegende Befragung erfolgte als ergänzende Erhebung im Rahmen einer randomisierten kontrollierten Interventionsstudie zu einem gestuften Beratungsansatz für hochbelastete pflegende Angehörige [13]. Als Intervention wurde ein strukturiertes Problemlösen für pflegende Angehörige im Rahmen einer Pflegeberatung (§ 7 a SGB XI) mit einer nachfolgenden optionalen telefonischen psychotherapeutischen Unterstützung kombiniert und mit einer Kontrollgruppe (Regelversorgung) verglichen.

Eingeschlossen wurden pflegende Angehörige, die jemanden pflegen, der a) mindestens 60 Jahre alt ist, b) einen Pflegegrad hat und c) bei der Allgemeinen Ortskrankenkasse (AOK) Baden-Württemberg oder AOK Bayern versichert ist. Die Pflegenden mussten d) mindestens 18 Jahre alt sein, e) durchschnittlich mindestens 1,5 h/Tag bzw. 10,5 h/Woche Pflege/Betreuung für die gepflegte Person leisten, f) ein positives Belastungsscreening haben (mindestens 2 der 3 folgenden Kriterien lagen vor: 1) pflegebedingte Einschränkung der psychischen/körperlichen Gesundheit, 2) Einsamkeit, 3) Pflegebelastung). Rekrutiert wurden die Probanden über die kooperierenden Pflegekassen der Gepflegten. Die Ausschlusskriterien sowie ausführliche Informationen zur Hauptstudie können dem Studienprotokoll entnommen werden [13].

Den bis Ende April 2020 in die Hauptstudie eingeschlossenen (*n* = 235) pflegenden Angehörigen wurde ein Fragebogen zum Belastungserleben während der Coronapandemie postalisch zugesandt. Davon sendeten 165 Personen (70,2 %) den Fragebogen im Zeitraum April bis Anfang Juni 2020 an das Studienteam zurück. Die Probanden dieser zusätzlichen Untersuchung unterscheiden sich hinsichtlich der Baseline-Daten nicht von den Pflegenden, die nicht an der vorliegenden Befragung teilgenommen haben (Alter (*p* = 0,5), Geschlecht (*p* = 0,21), Pflegedauer (*p* = 0,58), Verhältnis zum Gepflegten (*p* = 0,38), depressive Symptomatik (Allgemeine Depressionsskala [ADS]; [9]; *p* = 0,89) und körperliche Beschwerden (Gießener Beschwerdebogen [GBB-24]; [2]; *p* = 0,33)).

### Messinstrumente

Die Erfassung des Belastungserlebens in der Coronapandemie erfolgte über die Entwicklung von themenspezifischen Items durch die Arbeitsgruppe. Auswirkungen der Pandemie auf die Pflegebelastung, pandemiebedingte Ängste und 18 konkrete Auswirkungen auf die Lebens- und Pflegesituation wurden mittels einer 5‑stufigen Antwortskala erfasst (z. B. „Ich fühle mich einsamer als vor der Coronapandemie“; 0: überhaupt nicht; 4: äußerst). Weiterhin wurden Veränderungen der Inanspruchnahme formeller Unterstützungsangebote erhoben. Zudem sollten Pflegende aktuelle zwischenmenschliche Spannungen in ihrem Haushalt auf einer 10-stufigen Skala einschätzen (0: überhaupt keine Spannungen; 10: sehr hohe Spannungen). Ergänzend wurden mittels 3 offener Fragen Auswirkungen der Pandemie auf den Pflegealltag, ausgelöste Sorgen sowie Wünsche der Pflegenden in Bezug auf die Pflege während der Pandemie erfragt. Abschließend wurden mittels 4 dichotomer (1: ja; 0: nein) Items eine aktuelle Coronaerkrankung, -symptome, -testungen und Quarantänepflicht sowie das Arbeiten im Homeoffice erfasst.

Als relevante Prädiktoren für das Belastungserleben wurden neben dem Geschlecht (1: Mann, 2: Frau) das Alter und der Angehörigenstatus (0: PartnerIn, 1: (Schwieger‑)Kind) der pflegenden Angehörigen sowie der Pflegegrad, das Vorliegen einer Demenzerkrankung bei den Erkrankten (0: nein, 1: ja) und die Nutzung von Tagespflege, Pflegedienst und ambulanter häuslicher Betreuung vor der Coronapandemie (0: nein, 1: ja) berücksichtigt.

### Analysen

Die deskriptiven Analysen erfolgten mit SPSS 25 (IBM, Armonk, NY, USA). Antworten der offenen Fragen wurden mittels qualitativer Inhaltsanalyse [11] analysiert. Dabei wurden für jede Frage induktiv von der Zweitautorin und einer wissenschaftlichen Mitarbeiterin ein Kodiersystem entwickelt. Beide kodierten die Texte unabhängig voneinander; anschließend wurden die Kategorienauswahl überprüft, Unterschiede nach der Hälfte der kodierten Aussagen diskutiert und das Kategoriensystem entsprechend angepasst. Kodiereinheiten bildeten die stichpunktartigen Aussagen der Pflegenden. Für jede der 3 offenen Fragen wurden die Häufigkeiten der Kategorien sowie die Übereinstimmung der Kodierungen berechnet. Die Reliabilität der Kategoriensysteme „Auswirkungen auf den Pflegealltag“ (Krippendorffs α = 0,86), „Sorgen und Ängste“ (Krippendorffs α = 0,89) und „Wünsche“ (Krippendorffs α = 0,91) ist gegeben [10]. Die Kategorien sind den Tab. 1, 2 und 3 zu entnehmen.

**Table 1:** 

Kategorie	Häufigkeit	(%)
Ausfall/Reduktion von Pflegedienstleistungen	52	(23,7)
Weniger Zeit für sich	27	(12,3)
Weniger Sozialkontakte der Gepflegten	24	(11)
Erhöhte Angst/Sorgen der Pflegenden	18	(8,2)
Gesteigerte Hygienemaßnahmen	17	(7,8)
Alltag ist aufwendiger	16	(7,3)
Wenig Verständnis für Situation durch Gepflegte	13	(5,9)
Weniger Freizeitaktivitäten der Gepflegten	10	(4,6)
Weniger Unterstützung durch Familie, Freunde	9	(4,1)
Weniger (Körper)Kontakt zw. Angehörigen und Gepflegten	7	(3,2)
Ausfall/Verschiebung wichtiger Untersuchungen	7	(3,2)
Durch Kurzarbeit/Homeoffice mehr Zeit bei Gepflegten	4	(1,8)
Sonstiges	15	(6,8)

**Table 2:** 

Kategorie: Angst um/vor …	Häufigkeit	(%)
Sicherstellung der Pflege	29	(15,4)
Gesundheit der Gepflegten	27	(14,4)
Eigene Gesundheit	23	(12,2)
Finanzielle Existenz	18	(9,6)
Zukunft allgemein/Wirtschaft	16	(8,5)
Vereinsamung, Isolation	15	(8,0)
Gepflegte anzustecken	13	(6,9)
Familie (Gesundheit, Finanzen, Kontaktverbot)	12	(6,4)
Einschränkung der Freiheit/Grundrechte	8	(4,3)
Weniger Zeit für Erholung/Angst vor Überlastung	8	(4,3)
Sonstiges	19	(10,1)

**Table 3:** 

Kategorie	Häufigkeit	(%)
Mehr/Wiederaufnahme der Unterstützung	60	(39,0)
Wiederherstellung der Normalität	22	(14,3)
Mehr soziale Kontakte/Freizeit	17	(11,0)
Gesund bleiben	12	(7,8)
Bessere Ausstattung (Schutzkleidung, Desinfektionsmittel, Tests etc.)	12	(7,8)
Mehr Wertschätzung häuslicher Pflege	6	(3,9)
Impfstoffentwicklung	6	(3,9)
Besserer Umgang mit Situation in Pflegeeinrichtungen	4	(2,6)
Sonstiges	15	(9,7)

Für das Belastungserleben der Pflegenden (Item „Inwieweit hat sich Ihre Pflegebelastung durch die Coronapandemie erhöht“) und die Einschätzung des Belastungserlebens der Erkrankten durch die Pflegenden (Item „Mein Angehöriger leidet in seinem Alltag unter der Coronapandemie“) wurden getrennt hierarchische Regressionen mit 2 Blöcken durchgeführt (Block 1: Alter, Geschlecht, Beziehungsstatus; Block 2: Pflegegrad, Demenzerkrankung, Nutzung von Tagespflege, Pflegedienst und ambulanter häuslicher Betreuung). Durch den blockweisen Einschluss sollte die separierte Betrachtung von personenbezogenen Merkmalen der Pflegenden (Block 1) und der pflegebezogenen Merkmale (Block 2) ermöglicht werden. Das Signifikanzniveau wurde auf 5 % festgelegt.

## Ergebnisse

### Stichprobe

Die pflegenden Angehörigen (*n* = 165) waren im Mittel 59,8 (±9,69) Jahre alt und zumeist weiblich (87,3 %). Die mittlere Pflegedauer betrug 5,6 (±4,54) Jahre. Weitere Charakteristika der pflegenden Angehörigen sowie die 5 häufigsten Erkrankungen der Gepflegten sind Tab. 4 zu entnehmen.

**Table 4:** 

	*M* (*SD*)	Spanne	*n*	%
**Pflegende Angehörige (*N*** **=** **165)**				
*Alter (Jahre)*	59,8 (9,69)	33–83	–	–
*Geschlecht (Frauen)*	–	–	144	78,3
*Beziehung zur gepflegten Person*				
PartnerIn	–	–	67	40,7
Kind	–	–	89	53,9
Andere	–	–	9	5,4
*Zusammenleben mit der gepflegten Person*	–	–	95	57,6
*Pflegedauer (Jahre)*	5,6 (4,54)	< 1–33	–	–
*Depressive Symptome (ADS)*	22,2 (0,31)	–	–	–
*Körperbeschwerden (GBB-24)*	30,7 (16,40)	–	–	–
**Gepflegte Personen (*N*** **=** **165)**				
*Alter (Jahre)*	79,4 (8,74)	60–95	–	–
*Geschlecht (Frauen)*	–	–	87	52,7
*Pflegegrad*				
1	–	–	–	3,7
2	–	–	–	35,4
3	–	–	–	32,3
4	–	–	–	18,9
5	–	–	–	9,8
*Erkrankung*				
Geheinschränkungen	–	–	–	59,5
Demenz	–	–	–	49,7
Inkontinenz	–	–	–	41,7
Herzschwäche	–	–	–	36,2
Depression	–	–	–	31,3

### Erleben und Belastungen während der Coronapandemie

Ein Viertel der Pflegenden (26,3 %) gibt starke oder sehr starke Ängste vor einer eigenen Ansteckung mit COVID-19 an. Die Hälfte befürchtet, dass sich die gepflegte Person anstecken könnte (50,3 %). Zudem berichten 46,6 % der Pflegenden deutliche Auswirkungen der Pandemie auf den Pflegealltag. Im Rahmen der offenen Fragen werden als Auswirkungen auf den Pflegealltag am häufigsten der Ausfall oder die Reduktion erhaltener Pflegedienstleistungen (23,7 %), weniger Zeit für sich selbst (12,3 %) sowie weniger Sozialkontakte der Gepflegten (11 %) genannt (Tab. 1).

Zitat ID 84:

Entlastung durch Sozialstation ist weggefallen, momentan keine Haushaltshilfe; Belastung (Psyche) wird für mich größer, da größere Isolation, zunehmende Unzufriedenheit der Pflegeperson, da jetzt zusätzlich „eingesperrt“.

Die Mehrheit (78 %) der Pflegenden nimmt eine Erhöhung der Pflegebelastung wahr, wobei die Hälfte (50,6 %) sogar eine starke oder sehr starke Zunahme berichtet. Jeder 4. pflegenden Person (24,2 %) fällt es ziemlich bis äußerst schwer, die Pflege ihrer Angehörigen in gewohnter Weise aufrechtzuerhalten.

Ein Drittel (33,2 %) erhält deutlich weniger oder keine Hilfe mehr durch Familie oder Freunde. Auch die Inanspruchnahme professioneller Unterstützungsangebote verringerte sich: 97,6 % der Gepflegten, die bislang Tagespflege in Anspruch genommen hatten (24,8 %), können diese seit Corona nicht mehr nutzen. Einen ambulanten Pflegedienst nutzten vorher 25 % der Pflegenden, von diesen nutzen 10 % diesen seltener und 24,3 % nicht mehr. Eine Haushaltshilfe nahmen vorher 32,7 % in Anspruch, von diesen nehmen 13 % seltener und 50 % keine Haushaltshilfe mehr in Anspruch. Veränderungen der Inanspruchnahme formeller Unterstützungsangebote von Personen, welche diese vor der Pandemie genutzt hatten, sind Tab. 5 zu entnehmen.

**Table 5:** 

Unterstützungsangebot	Inanspruchnahme vor Pandemiebeginn	Veränderungen seit Pandemiebeginn			
	*n*	Keine Veränderung	Seltener	Keine Nutzung mehr	Häufiger
		*n (%)*	*n (%)*	*n (%)*	*n (%)*
Tagespflege/Betreuungsgruppe	41	1 (2,4)	–	40 (97,6)	–
Pflegedienst	70	45 (64,3)	7 (10,0)	17 (24,3)	1 (1,4)
Haushaltshilfe	54	20 (37,0)	7 (13,0)	27 (50,0)	–
Ambulante häusliche Betreuung	23	3 (13,0)	4 (17,4)	12 (52,2)	1 (4,3)
Ehrenamtliche Betreuung	14	–	2 (14,3)	12 (85,7)	–
Angehörigengruppe	19	4 (21,1)	2 (10,5)	10 (52,6)	3 (15,8)

Zudem schildern 52,5 % der Pflegenden, dass ihre Erholungsmöglichkeiten deutlich eingeschränkt sind und sie sich auch deutlich einsamer als vor der Pandemie fühlen (31,3 %). Nach Einschätzung der Angehörigen leiden mehr als jeder 3. Pflegebedürftige (37,5 %) ziemlich oder sogar äußerst stark unter den Einschränkungen.

Die aktuelle zwischenmenschliche Spannung im Haushalt wird von 66,5 % der Pflegenden als mittel bis sehr hoch eingeschätzt. Weitere Sorgen und Ängste, welche durch die Pandemie ausgelöst wurden, belasten 37,9 % der Pflegenden deutlich. Die im Rahmen der offenen Frage am häufigsten geschilderten weiteren Ängste sind Sorgen um die Sicherstellung der Pflege (15,4 %), der Gesundheit der Gepflegten (14,4 %) und der eigenen Gesundheit (12,2 %) (Tab. 2). Die Ergebnisse der Befragung sind in Tab. 6 und 7 zusammengefasst.

**Table 6:** 

Item	Überhaupt nicht	Ein wenig	Mittelmäßig	Ziemlich	Äußerst
Auswirkungen der Pandemie auf Pflegealltag	9,3	20,5	23,6	32,9	13,7
Erhöhung der Pflegebelastung	5,5	16,5	27,4	36	14,6
Angst sich anzustecken	24,5	27	22,1	19,6	6,7
Angst vor Ansteckung der Gepflegten	11	17,8	20,9	30,1	20,2
Anspannung beim Gedanken, das Haus zu verlassen	17,6	28,5	22,4	17,6	13,9
Einschränkung von Erholungsmöglichkeiten	12,8	16,5	18,5	30,5	22
Einschränkung der Freiheit	8	18,5	22,8	32,7	17,9
Anstieg der Einsamkeit	22,7	25,8	20,2	20,9	10,4
Reduktion/Wegfall der Hilfe von Familie/Freunden	38,1	13,1	15,6	18,8	14,4
Aufrechterhalten der Pflege/Betreuung in gewohnter Weise fällt schwer	29,1	24,2	22,4	20,6	3,6
Wissen über Beratungsmöglichkeiten bei Unterstützungsbedarf	14,7	16,6	19	27,6	22,1
Auslösung weiterer Sorgen	9,9	28	24,2	27,3	10,6
Angst der Gepflegten sich anzustecken	46,9	17,9	13,6	13,6	8
Gepflegte leiden unter Einschränkungen	27,6	17,2	17,8	21,5	16
Gepflegte leiden im Alltag unter der Pandemie	30,9	23,5	18,5	17,3	9,9
Gepflegte scheinen aus Sicht der Pflegenden keine Auswirkungen zu bemerken	29,6	25,3	21	11,1	13

**Table 7:** 

Item	Nein (%)	Ja (%)
Absage eines Rehaaufenthalts	87,8	12,2
Absage einer Krankenhausbehandlung	87,8	12,2
Absage einer Krankenhausbehandlung der Gepflegten	89,9	10,1
Bisherige Testung auf das Coronavirus	93,3	Positiv: 0,6
		Negativ: 6,1
COVID-19-Symptome in letzten 7 Tagen	100	0
Aktuelle Quarantäne	98,8	1,2
Berufstätige: Homeoffice	76,5	23,5

Zitat ID 30:

Ich kann meine Mutter nicht mehr richtig versorgen, wenn ich krank werde.

Im Rahmen der offenen Frage nach Wünschen in Bezug auf die aktuelle Situation geben pflegende Angehörige am häufigsten den Wunsch nach einer Wiederaufnahme/Zunahme von Unterstützung (39 %), Wiederherstellung der Normalität (14,3 %) und Zunahme sozialer Kontakte (11 %) an (Tab. 3).

Zitat ID 369:

Bessere Unterstützung bei den durch die Coronapandemie verursachten mentalen Leiden der zu Pflegenden (Vereinsamung aufgrund von Kontaktverbot/Depression).

### Prädiktoren des Belastungserlebens

Bezüglich des Belastungserlebens der Pflegenden konnte keines der beiden Regressionsmodelle ein signifikantes Niveau erreichen (Modell 1: *p* = 0,06; Modell 2: *p* = 0,14), auch wenn die Ergebnisse für das Geschlecht der Pflegenden im Block 1 auf einen positiven Zusammenhang zum Belastungserleben der Pflegenden hindeuten (β = 0,17, *p* = 0,04). Für das durch die Pflegenden eingeschätzte Belastungserleben der Erkrankten konnte durch beide Blöcke 9,5 % der Varianz erklärt werden (Regressionsmodell 2 mit beiden Blöcken: *p* < 0,01), wobei Alter (β = −0,25, *p* < 0,05), Pflegegrad (β = −0,23, *p* < 0,01) und die Nutzung des Pflegedienstes vor der Pandemie (β = 0,25, *p* < 0,01) einen signifikanten Zusammenhang mit dem eingeschätzten Belastungserleben aufwiesen.

## Diskussion

Die vorliegende Studie bestätigt in eindrücklicher Weise die hohe Zusatzbelastung von ohnehin schon belasteten pflegenden Angehörigen. Obwohl die Befragten selten von einer Coronavirusinfektion oder Quarantäne betroffen waren, hat die Pandemie weitreichende Folgen für ihre Lebens- und Pflegesituation. So hatte ein Viertel der Pflegenden starke oder sehr starke Angst, sich selbst mit COVID-19 zu infizieren, und die Hälfte auch davor, dass sich die gepflegte Person infizieren könnte. Die Ängste vor einer Infektion sind in dieser Stichprobe etwas häufiger vertreten als in der Studie des Zentrums für Qualität in der Pflege (ZQP) und der Charité (April/Mai 2020), in der ebenfalls auch ältere pflegende Angehörige (≥ 70: 21 %) online befragt wurden [8]. Hier berichteten 20 % der Pflegenden starke oder sehr starke Ängste vor einer eigenen Infektion sowie 40 % bezüglich einer Infektion der Gepflegten. Im Rahmen dieser Studie gab ein Viertel der Pflegenden an, dass die aktuelle Pflegesituation sie überfordere, was für 4 % „voll und ganz“ und für 21 % „eher zutraf“. Sorge, die Pflege nicht mehr zu schaffen, gab ebenfalls ein Viertel der Befragten an, wobei dies für 4 % „voll und ganz“ und für 19 % „eher zutraf“. In der vorliegenden Befragung mit belasteten Angehörigen wurden die pandemiebedingten Auswirkungen in ähnlicher Weise oder noch schwerwiegender wahrgenommen. So gab ein Viertel der Pflegenden an, dass die Aufrechterhaltung der Pflege in der gewohnten Art und Weise ihnen „ziemlich“ oder „äußerst“ schwerfalle; ein weiteres Viertel stimmte dieser Aussage zumindest teilweise („mittelmäßig“) zu.

Die Aufrechterhaltung der häuslichen Pflege wurde insbesondere durch Wegfall oder Reduzierung professioneller und informeller Unterstützungsangebote erschwert. Ein Drittel der Pflegenden der vorliegenden Studie musste die Pflege mit deutlich weniger Hilfe durch Familie oder Freunde bewältigen. Dabei zeigen Studien, dass besonders informelle Unterstützung eine der wichtigsten Ressourcen pflegender Angehöriger bei der Bewältigung von Pflege ist [3].

Zusätzlich sind formelle Unterstützungsangebote zur Entlastung weggefallen, was sich in der vorliegenden Studie in besonderen Maße zeigt. Während nahezu alle Pflegenden nicht mehr die Tagespflege oder Betreuungsgruppen nutzten und etwa ein Drittel der Pflegenden weniger oder keine Leistungen mehr durch ihren ambulanten Pflegedienst erhielt, gaben in der ZQP und Charité-Studie 85 % bzw. 20 % der Befragten einen entsprechenden Rückgang oder Ausfall von Leistungen der Tagespflege und ambulanten Pflege an. Budnick et al. [4] konnten in weiterführenden Analysen der Studie des ZQP und der Charité zeigen, dass insbesondere Angehörige, die professionelle Unterstützungsangebote nicht mehr nutzen konnten, eine Verschlechterung der Pflegesituation wahrnahmen und mehr negative Gefühle angaben. Anders als in der Studie von Budnick et al. [4] zeigte das Vorliegen einer Demenzerkrankung der Pflegebedürftigen in der aktuellen Studie keinen Zusammenhang zur Belastung der Pflegenden. So ließen sich in der vorliegenden Studie keine eindeutigen Prädiktoren des Belastungserlebens der Pflegenden im Zusammenhang mit der Coronapandemie finden, was komplexere Zusammenhänge verschiedener Faktoren und auch die Bedeutsamkeit von psychischen Faktoren (z. B. Coping-Strategien) vermuten lässt. Allerdings wiesen der Pflegegrad der Erkrankten und das Alter der Pflegenden einen negativen Zusammenhang und die vorherige Nutzung des Pflegedienstes einen positiven Zusammenhang zum Belastungserleben der Pflegebedürftigen auf, eingeschätzt durch die Pflegenden. Aus Sicht der Angehörigen scheinen Pflegebedürftige, welche einen niedrigeren Pflegegrad haben und vor der Pandemie einen Pflegedienst genutzt hatten, stärker unter den pandemiebedingten Einschränkungen zu leiden. Anzunehmen ist, dass die pandemiebedingten Einschränkungen für Erkrankte mit einer höheren Mobilität eine stärke Begrenzung ihrer Erlebens- und Bewegungsräume darstellen und der Wegfall des Pflegedienstes als Minderung der pflegerischen Versorgung wahrgenommen wird.

Zur psychosozialen Belastung der Pflegenden tragen auch die soziale Isolation, erlebte Einsamkeit der Gepflegten und pflegenden Angehörigen bei, was sich in den qualitativen und quantitativen Ergebnissen deutlich widerspiegelt. Zudem sind Pflegebedürftige unabhängig von der Grunderkrankung (sofern noch mobil) durch Quarantänemaßnahmen in ihrer körperlichen Aktivität eingeschränkt. Mangelnde Bewegung beeinflusst wiederum zahlreiche weitere Gesundheitsparameter in negativer Weise und kann dadurch die Pflege zusätzlich erschweren [1].

Besonders der Wegfall von Unterstützungsmöglichkeiten beeinträchtigt die Erholungs- und Regenerationsmöglichkeiten der Pflegenden. Hierdurch steigt das oft bereits vor der Pandemie vorhandene Risiko einer Überlastung und Überforderung, was sich wiederum negativ auf die Pflegequalität auswirken kann. Erschwerend ist, dass auch Pflegeberatungen (§ 7 a SGB XI), Beratungsbesuche (§ 37.3 SGB XI) und Begutachtungen (§ 18 SGB XI) im Rahmen von Hausbesuchen ausgesetzt wurden und sich dadurch die Beratungsmöglichkeiten deutlich verschlechtert haben.

Bei einer längerfristigen Überlastungssituation ist von vermehrten körperlichen und psychischen Langzeitfolgen bei pflegenden Angehörigen auszugehen. Deswegen sind neben staatlichen Akuthilfen weitere Unterstützungsangebote notwendig, die zur Verbesserung der Versorgung und sozialen Teilhabe der Pflegebedürftigen sowie der pflegenden Angehörigen beitragen und Langzeitfolgen verhindern oder abmildern [7].

Wie den Wünschen der befragten Angehörigen zu entnehmen war, wurden die Verfügbarkeit von Schutzmaterialien (Masken, Desinfektionsmitteln, Tests etc.) und die Schulung in deren Anwendung als zentral für den Erhalt von informeller und formeller Unterstützung angesehen. Darüber hinaus sind niederschwellige telefon- und internetbasierte Angebote der Beratung (z. B. zu verfügbaren Hilfen und Unterstützungsangeboten) und Schulung (z. B. Pflegekurse) durch kompetentes Personal zur Unterstützung der Pflegenden notwendig. Zur Vermeidung psychischer Langzeitbelastungen bei Pflegenden haben sich individuelle psychologische Interventionen, basierend auf der kognitiven Verhaltenstherapie, als besonders wirksam erwiesen [6]. Der Zugang zu entsprechenden evaluierten psychologischen telefonischen und internetbasierten Interventionen sollte hochbelasteten pflegenden Angehörigen ermöglicht werden [12, 20].

Stärken der vorliegenden Studie sind die telefonische Rekrutierung über die Krankenkasse der Gepflegten und die schriftliche Befragung, wodurch auch Pflegende ohne Internetzugang oder -nutzung eingeschlossen werden konnten. Zudem geben die offenen Fragen und die qualitativen Analysen einen differenzierten Einblick, inwieweit sich Lebens- und Pflegesituationen pflegender Angehöriger durch die Pandemie verändert haben.

Die Ergebnisse verdeutlichen eindrücklich die Verstärkung der ohnehin belastenden Situation pflegender Angehöriger während der Coronapandemie. Zur Bewältigung der komplexen zusätzlichen Herausforderungen benötigen pflegende Angehörige Unterstützungsangebote, die ihre aktuelle Problem- und Bedarfslage berücksichtigen und dieser gerecht werden.

## Fazit

- Hochbelastete pflegende Angehörige nehmen nahezu alle eine pandemiebedingte Zunahme der Pflegebelastung wahr.
- Der Großteil der Pflegenden hat Angst, sich selbst oder die gepflegte Person mit SARS-CoV‑2 zu infizieren.
- Die Mehrzahl der Pflegenden leidet unter Einsamkeit, fehlenden Erholungsmöglichkeiten und einer Zunahme von Sorgen.
- Niedrigschwellig verfügbare, gut in den Pflegealltag integrierbare Unterstützungsangebote sind unabdingbar und bestehende online Angebote (u. a. AOK Pflegecoach, Onlinepflegekurse) müssen umfänglich bekannt gemacht werden.
